# Spectrum of Beta-Lactamase Inhibition by the Cyclic Boronate QPX7728, an Ultrabroad-Spectrum Beta-Lactamase Inhibitor of Serine and Metallo-Beta-Lactamases: Enhancement of Activity of Multiple Antibiotics against Isogenic Strains Expressing Single Beta-Lactamases

**DOI:** 10.1128/AAC.00212-20

**Published:** 2020-05-21

**Authors:** Olga Lomovskaya, Ruslan Tsivkovski, Kirk Nelson, Debora Rubio-Aparicio, Dongxu Sun, Maxim Totrov, Michael N. Dudley

**Affiliations:** a Qpex Biopharma, Inc., San Diego, California, USA; b Molsoft L.L.C., San Diego, California, USA

**Keywords:** serine beta-lactamases, metallo-beta-lactamases, QPX7728, beta-lactamase inhibitor

## Abstract

QPX7728 is an ultrabroad-spectrum boronic acid beta-lactamase inhibitor, with potent inhibition of key serine and metallo-beta-lactamases being observed in biochemical assays. Microbiological studies using characterized strains were used to provide a comprehensive characterization of the spectrum of beta-lactamase inhibition by QPX7728. The MICs of multiple antibiotics administered intravenously only (ceftazidime, piperacillin, cefepime, ceftolozane, and meropenem) and orally bioavailable antibiotics (ceftibuten, cefpodoxime, tebipenem) alone and in combination with QPX7728 (4 μg/ml), as well as comparator agents, were determined against panels of laboratory strains of Pseudomonas aeruginosa and Klebsiella pneumoniae expressing over 55 diverse serine and metallo-beta-lactamases. QPX7728 significantly enhanced the potency of antibiotics against strains expressing class A extended-spectrum beta-lactamases (CTX-M, SHV, TEM, VEB, PER) and carbapenemases (KPC, SME, NMC-A, BKC-1), consistent with the beta-lactamase inhibition demonstrated in biochemical assays. It also inhibited both plasmidic (CMY, FOX, MIR, DHA) and chromosomally encoded (P99, PDC, ADC) class C beta-lactamases and class D enzymes, including carbapenemases, such as OXA-48 from *Enterobacteriaceae* and OXA enzymes from Acinetobacter baumannii (OXA-23/24/72/58). QPX7728 is also a potent inhibitor of many class B metallo-beta-lactamases (NDM, VIM, CcrA, IMP, and GIM but not SPM or L1). Addition of QPX7728 (4 μg/ml) reduced the MICs for a majority of the strains to the level observed for the control with the vector alone, indicative of complete beta-lactamase inhibition. The ultrabroad-spectrum beta-lactamase inhibition profile makes QPX7728 a viable candidate for further development.

## Abstract (precis)

QPX7728 is an ultrabroad-spectrum boronic acid beta-lactamase inhibitor, with potent inhibition of key serine and metallo-beta-lactamases being observed in biochemical assays. Microbiological studies using characterized strains were used to provide a comprehensive characterization of the spectrum of beta-lactamase inhibition by QPX7728. The MICs of multiple antibiotics administered intravenously only (ceftazidime, piperacillin, cefepime, ceftolozane, and meropenem) and orally bioavailable antibiotics (ceftibuten, cefpodoxime, tebipenem) alone and in combination with QPX7728 (4 μg/ml), as well as comparator agents, were determined against panels of laboratory strains of Pseudomonas aeruginosa and Klebsiella pneumoniae expressing over 55 diverse serine and metallo-beta-lactamases.

## INTRODUCTION

The development of beta-lactamase inhibitors (BLIs) in combination with beta-lactams is a powerful strategy to protect beta-lactams from beta-lactamase-mediated hydrolysis and preserve the clinical utility of this widely used group of antimicrobial agents. Four beta-lactam–BLI combination agents that were recently approved by the FDA are ceftolozane-tazobactam (approved in 2014), ceftazidime-avibactam (approved in 2015), meropenem-vaborbactam (approved in 2017), and imipenem-relebactam (approved in 2019); all of them represent significant progress in addressing serious drug-resistant Gram-negative bacterial infections (1–4). Avibactam and relebactam, which are diazabicyclooctane derivatives (DBOs), and vaborbactam, which is a cyclic boronate, are dual inhibitors of class A and class C beta-lactamases, and all three are potent inhibitors of KPC (5–7); however, none of these BLIs inhibit either class D carbapenemases in Acinetobacter baumannii or metallo-beta-lactamases (MBLs). While these combination products represent advances for the treatment of infections caused by pathogens that are recognized to be urgent or serious threats by the CDC, many pathogens and resistance mechanisms remain unaddressed (8).

**FIG 1:**
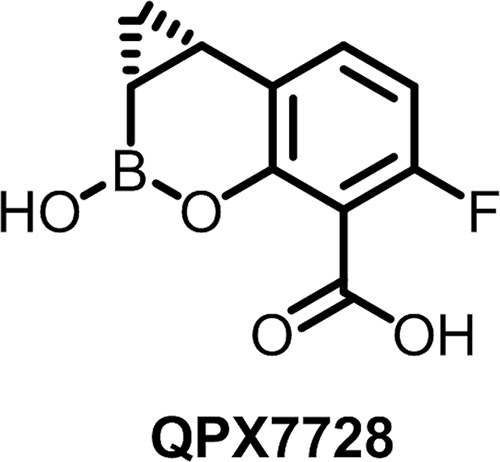
Structure of QPX7728.

QPX7728 (Fig. 1) is a beta-lactamase inhibitor (BLI) which emerged from the boronic acid pharmacophore program that led to vaborbactam (9), the first FDA- and EMA-approved agent from this new class. As part of the drug discovery program that was a follow-on to the program that led to vaborbactam, we identified lead compounds with an inhibition profile broader than that of vaborbactam that included other serine enzymes and, notably, metallo-beta-lactamases from class B. An extensive medicinal chemistry program guided by our early lead compounds and structure-based design culminated in the discovery of QPX7728 (10). In biochemical studies using purified beta-lactamases (11), we demonstrated that QPX7728 is a potent inhibitor of several prevalent serine and metallo-beta-lactamases (12–14), with 50% inhibitory concentration (IC_50_) values generally being in the low-nanomolar range. These biochemical results (11) show that QPX7728 inhibits the broadest spectrum of beta-lactamases among the marketed BLIs and those in various stages of clinical development (15, 16). This paper provides further details on the spectrum of beta-lactamase inhibition of panels of isogenic strains expressing single beta-lactamases by QPX7728 in combination with multiple different beta-lactam antibiotics.

## RESULTS AND DISCUSSION

To further characterize the spectrum of inhibition by QPX7728, we employed a widely used microbiological approach of studying bacterial strains of isogenic backgrounds expressing individual beta-lactamases and determining the MICs of beta-lactams and beta-lactam–beta-lactamase inhibitor (BLI) combinations (17, 18). This allows the efficient expansion of information on the beta-lactamase inhibition profile obtained in enzyme inhibition experiments, allows correlation of cellular potency with the available enzyme inhibition data, and generates data on the whole-cell antibiotic potentiation activity in the presence or absence of mechanisms that alter permeability or efflux. We used panels of engineered strains of Pseudomonas aeruginosa (host strain PAM1154, which lacks efflux pumps) and Klebsiella pneumoniae (host strain KPM1001 [ATCC 43816], which is a wild-type strain) expressing over 55 diverse beta-lactamases. The use of P. aeruginosa as an isogenic background facilitates the detection of the beta-lactamase activity (as an MIC increase) of low-catalytic-efficiency enzymes that rely heavily on the low permeability of the outer membrane (19). The use of strains that lack efflux pumps ensures appropriate interpretation of the extent of inhibition in whole-cell systems. Another objective of the study was to assess whether the level of potentiation by QPX7728 depended on the partner beta-lactam.

The MICs of ceftazidime, piperacillin, cefepime, ceftolozane, and meropenem (antibiotics administered intravenously [i.v.] only) alone and in combination with QPX7728 against the panel of P. aeruginosa strains were determined (Tables 1 and 2). Since QPX7728 can be delivered by i.v. or oral administration (10), the MICs of ceftibuten, cefpodoxime, and tebipenem (orally bioavailable antibiotics) alone or in combination with QPX7728 against the panel of K. pneumoniae were determined (Table 3). For the purposes of these experiments, the concentration of QPX7728 tested in these microbiological assays was arbitrarily set at 4 μg/ml, solely for the purpose of comparing the activity QPX7728 in combination with various antibiotics against different beta-lactamases. Future dose-response studies, pharmacokinetic (PK)-pharmacodynamic (PD) studies in models of infection, and studies of PK in humans will ultimately be used to provide a rationale for the selection of a testing concentration of QPX7728 and to propose a specific protocol for *in vitro* susceptibility testing for clinical use. Avibactam, relebactam, and vaborbactam were used as comparator BLIs and were also tested at a fixed concentration of 4 μg/ml.

**TABLE 1:** MICs of ceftazidime and piperacillin alone and in combination with BLIs against the panel of engineered P. aeruginosa strains producing various cloned beta-lactamases^*a*^

Strain	Beta-lactamase	Class	MIC (μg/ml)									
			Ceftazidime					Piperacillin				
			Alone	With BLIs				Alone	With BLIs			
				AVI	RELE	VAB	QPX7728		AVI	RELE	VAB	QPX7728
PAM4175	pUCP24 vector	None	**0.25**	0.25	0.25	0.25	≤0.06	**0.125**	0.125	≤0.06	0.125	≤0.06
PAM4819	CTX-M-2	A	**1**	0.125	0.125	0.25	≤0.06	**64**	≤0.06	≤0.06	0.125	≤0.06
PAM4743	CTX-M-15	A	**32**	0.25	0.25	0.25	0.125	**>64**	0.125	0.25	0.5	≤0.06
PAM4820	CTX-M-25	A	**8**	0.25	0.25	0.25	0.125	**>64**	0.25	0.5	4	≤0.06
PAM4822	CTX-M-27	A	**64**	0.25	0.5	0.5	0.125	**>64**	0.25	1	2	≤0.06
PAM4886	GES-1	A	**64**	0.25	0.5	0.5	0.25	**4**	0.25	0.125	0.125	≤0.06
PAM4800	GES-19	A	**>64**	0.5	2	2	0.125	**8**	0.25	0.125	0.25	≤0.06
PAM4840	OXY-6-2	A	**0.5**	0.25	0.25	0.5	0.25	**>64**	2	0.25	>32	≤0.06
PAM4842	PER-2	A	**>64**	0.5	0.25	1	0.125	**1**	0.125	0.125	0.125	≤0.06
PAM4907	PER-4	A	**>64**	>64	32	16	0.25	**0.5**	0.25	0.25	0.125	≤0.06
PAM4874	SHV-12	A	**>64**	0.25	2	8	0.25	**>64**	0.5	1	8	≤0.06
PAM4878	TEM-10	A	**>64**	0.25	2	8	0.125	**32**	0.125	0.5	8	≤0.06
PAM4908	VEB-1	A	**>64**	1	1	4	0.25	**8**	0.25	0.125	0.25	≤0.06
PAM4910	VEB-2	A	**>64**	1	1	8	0.25	**16**	0.25	0.125	0.25	≤0.06
PAM4912	VEB-3	A	**>64**	1	1	4	0.25	**8**	0.25	0.125	0.25	≤0.06
PAM4938	VEB-9	A	**256**	2	2	8	≤0.25	**16**	0.13	0.125	0.25	≤0.06
PAM4135	KPC-2	A-CRB	**16**	0.25	0.25	0.25	0.125	**>64**	1	0.5	0.125	≤0.06
PAM4689	KPC-3	A-CRB	**>64**	0.5	0.5	0.25	0.125	**>64**	1	0.5	0.125	≤0.06
PAM4794	NMC-1	A-CRB	**1**	0.25	0.25	0.25	0.125	**16**	0.5	0.5	0.125	≤0.06
PAM4864	SFC-1	A-CRB	**2**	0.25	0.25	0.25	0.125	**>64**	0.25	1	0.125	≤0.06
PAM4801	GES-20	A-CRB	**4**	0.25	0.5	0.25	0.125	**16**	0.25	0.125	0.125	≤0.06
PAM4744	SME-2	A-CRB	**0.5**	0.25	0.25	0.25	0.125	**8**	0.5	0.25	0.125	≤0.06
PAM4938	VCC-1	A-CRB	**ND**	ND	ND	ND	ND	**16**	≤0.06	0.25	≤0.06	≤0.06
PAM4676	BKC-1	A-CRB	**32**	0.25	0.5	0.25	0.125	**32**	0.5	0.5	0.25	≤0.06
PAM4186	CMY-2	C	**16**	0.25	0.25	0.25	0.125	**8**	≤0.06	≤0.06	0.125	≤0.06
PAM4825	MIR-1	C	**16**	0.25	0.25	0.25	0.125	**8**	0.125	0.125	0.125	≤0.06
PAM4745	P99 (ECL chAmpC)	C	**64**	0.25	0.25	0.5	0.125	**64**	0.125	0.125	0.5	≤0.06
PAM4869	PDC-1 (Pa chAmpC)	C	**16**	0.25	0.25	1	0.125	**>64**	0.5	0.5	8	≤0.06
PAM4884	ADC-181 (AB chAmpC)	C	**8**	0.5	0.25	8	0.25	**1**	0.125	≤0.06	1	≤0.06
PAM4827	OXA-1	D	**0.125**	0.125	0.125	0.125	≤0.06	**4**	≤0.06	0.5	4	≤0.06
PAM4792	OXA-2	D	**8**	0.25	0.5	8	0.125	**64**	0.5	1	64	≤0.06
PAM4846	OXA-9	D	**0.25**	0.25	0.25	0.25	0.125	**8**	0.125	4	0.25	≤0.06
PAM4790	OXA-10	D	**0.25**	0.25	0.25	0.25	0.125	**0.125**	0.125	≤0.06	0.125	≤0.06
PAM4217	OXA-48	D-CRB	**0.25**	0.25	0.25	0.25	0.125	**16**	0.125	8	8	≤0.06
PAM4875	OXA-23	D-CRB	**0.25**	0.25	0.25	0.25	0.125	**16**	4	>32	16	≤0.06
PAM4876	OXA-72	D-CRB	**0.25**	0.25	0.25	0.25	0.125	**16**	4	8	16	≤0.06
PAM4877	OXA-58	D-CRB	**0.25**	0.25	0.25	0.25	0.125	**4**	1	4	4	≤0.06
PAM4179	NDM-1	B	**>64**	>64	>64	>64	32	**64**	64	64	64	0.25
PAM4917	NDM-7	B	**>64**	>64	>64	>64	32	**32**	32	32	32	0.25
PAM4795	VIM-1	B	**64**	64	64	64	0.25	**16**	16	32	16	≤0.06
PAM4798	VIM-2	B	**16**	16	32	16	0.125	**16**	16	16	32	≤0.06
PAM4881	VIM-7	B	**0.5**	0.25	1	0.5	0.25	**2**	2	2	2	≤0.06
PAM4887	IMP-1	B	**>64**	>64	>64	>64	2	**1**	0.5	0.5	0.5	≤0.06
PAM4888	IMP-4	B	**>64**	>64	>64	>64	2	**0.5**	0.5	0.5	0.5	≤0.06
PAM4196	IMP-13	B	**64**	64	64	64	2	**0.5**	0.5	0.5	0.5	≤0.06
PAM4198	IMP-15	B	**64**	64	64	64	1	**0.5**	0.25	0.25	0.5	≤0.06
PAM4890	IMP-19	B	**>64**	>64	>64	>64	4	**0.25**	0.25	0.25	0.25	≤0.06
PAM4889	IMP-26	B	**>64**	>64	>64	>64	64	**0.125**	0.125	≤0.06	0.125	≤0.06
PAM4879	CcrA	B	**4**	4	4	4	0.125	**0.25**	0.125	0.125	0.25	≤0.06
PAM4883	GIM-1	B	**>64**	64	64	64	0.5	**>64**	>64	>64	>64	2
PAM4885	SPM-1	B	**>64**	>64	>64	>64	>64	**32**	32	32	32	8
PAM4880	L1	B	**64**	64	64	64	64	**32**	32	32	32	8

a 1A-CRB, class A carbapenemase; 2D-CRB, class D carbapenemase; BLIs, beta-lactamase inhibitors; VAB, vaborbactam; AVI, avibactam; RELE, relebactam; ND, not determined. All BLIs were tested at a fixed concentration of 4 μg/ml. Boldface indicates MIC to antibiotic alone.

**TABLE 2:** MICs of cefepime, ceftolozane, and meropenem alone and in combination with BLIs against the panel of engineered P. aeruginosa strains producing various cloned beta-lactamases^*a*^

Strain	Beta-lactamase	MIC (μg/ml)								
		Cefepime			Ceftolozane			Meropenem		
		Alone	With BLIs		Alone	With BLIs		Alone	With BLIs	
			AVI	QPX7728		AVI	QPX7728		AVI	QPX7728
PAM4175	vector	**0.125**	≤0.06	≤0.06	**0.25**	0.25	0.125	**≤0.06**	≤0.06	≤0.06
PAM4819	CTX-M-2	**>64**	0.25	≤0.06	**4**	0.125	≤0.06	**0.125**	0.125	≤0.06
PAM4743	CTX-M-15	**>64**	0.25	≤0.06	**64**	0.25	0.125	**0.5**	≤0.06	≤0.06
PAM4820	CTX-M-25	**2**	0.125	≤0.06	**16**	0.25	0.125	**0.5**	≤0.06	≤0.06
PAM4822	CTX-M-27	**32**	0.125	≤0.06	**64**	0.25	0.25	**0.5**	≤0.06	≤0.06
PAM4886	GES-1	**1**	0.125	≤0.06	**32**	0.5	0.125	**0.25**	≤0.06	0.125
PAM4800	GES-19	**0.5**	≤0.06	≤0.06	**>64**	0.5	0.125	**1**	≤0.06	0.125
PAM4840	OXY-6-2	**>32**	0.25	≤0.06	**1**	0.25	0.125	**0.25**	≤0.06	≤0.06
PAM4842	PER-2	**>64**	0.25	≤0.06	**>64**	1	0.125	**0.125**	≤0.06	≤0.06
PAM4907	PER-4	**32**	≤0.06	≤0.06	**32**	32	≤0.06	**0.125**	0.125	≤0.06
PAM4874	SHV-12	**>64**	0.125	≤0.06	**16**	0.25	0.125	**0.25**	≤0.06	≤0.06
PAM4878	TEM-10	**>64**	0.125	≤0.06	**16**	0.25	0.125	**0.25**	≤0.06	≤0.06
PAM4908	VEB-1	**>64**	0.125	≤0.06	**>64**	0.5	≤0.06	**0.25**	≤0.06	≤0.06
PAM4910	VEB-2	**16**	0.125	≤0.06	**>64**	0.5	≤0.06	**0.25**	≤0.06	≤0.06
PAM4912	VEB-3	**64**	0.25	≤0.06	**>64**	0.5	≤0.06	**0.5**	≤0.06	≤0.06
PAM4938	VEB-9	**0.5**	0.125	≤0.06	**256**	2	≤0.25	**0.13**	≤0.06	≤0.06
PAM4135	KPC-2	**4**	0.125	≤0.06	**8**	0.25	0.125	**64**	≤0.06	≤0.06
PAM4689	KPC-3	**32**	0.25	≤0.06	**32**	0.5	0.125	**64**	≤0.06	≤0.06
PAM4794	NMC-1	**64**	0.125	≤0.06	**0.5**	0.25	0.125	**64**	0.125	≤0.06
PAM4864	SFC-1	**16**	≤0.06	≤0.06	**2**	0.25	0.125	**64**	0.125	≤0.06
PAM4801	GES-20	**>64**	8	≤0.06	**4**	0.5	0.25	**16**	≤0.06	≤0.06
PAM4744	SME-2	**>64**	0.25	≤0.06	**0.5**	0.25	0.125	**16**	≤0.06	≤0.06
PAM4938	VCC-1	**>64**	0.25	≤0.06	ND	ND	ND	**8**	≤0.06	≤0.06
PAM4676	BKC-1	**128**	2	≤0.25	**2**	0.25	0.125	**4**	≤0.06	≤0.06
PAM4186	CMY-2	**1**	≤0.06	≤0.06	**2**	0.25	0.125	**0.25**	≤0.06	≤0.06
PAM4825	MIR-1	**2**	0.125	≤0.06	**4**	0.25	0.125	**0.5**	≤0.06	≤0.06
PAM4745	P99 (ECL chAmpC)	**8**	0.125	≤0.06	**8**	0.25	0.125	**0.5**	≤0.06	≤0.06
PAM4869	PDC-1 (Pa chAmpC)	**4**	0.125	≤0.06	**1**	0.25	0.125	**0.5**	≤0.06	≤0.06
PAM4884	ADC-181 (AB chAmpC)	**0.5**	≤0.06	≤0.06	**2**	0.25	0.125	**0.125**	≤0.06	≤0.06
PAM4827	OXA-1	**1**	≤0.06	≤0.06	**0.125**	0.125	≤0.06	**≤0.06**	≤0.06	≤0.06
PAM4792	OXA-2	**0.5**	0.125	≤0.06	**8**	0.25	0.125	**2**	0.5	≤0.06
PAM4846	OXA-9	**1**	0.125	≤0.06	**0.5**	0.25	0.125	**0.25**	≤0.06	≤0.06
PAM4790	OXA-10	**0.125**	≤0.06	≤0.06	**0.25**	0.25	0.125	**0.125**	≤0.06	≤0.06
PAM4217	OXA-48	**0.25**	≤0.06	≤0.06	**0.25**	0.25	0.125	**4**	≤0.06	≤0.06
PAM4875	OXA-23	**2**	0.25	≤0.06	**0.25**	0.25	0.125	**2**	0.5	≤0.06
PAM4876	OXA-72	**2**	0.25	≤0.06	**0.25**	0.25	0.125	**2**	1	≤0.06
PAM4877	OXA-58	**0.125**	≤0.06	≤0.06	**0.25**	0.25	0.125	**0.25**	≤0.06	≤0.06
PAM4179	NDM-1	**>64**	>64	2	**>64**	>64	32	**32**	32	1
PAM4917	NDM-7	ND	ND	ND	**>64**	>64	32	**32**	32	1
PAM4795	VIM-1	**64**	64	0.25	**>64**	>64	1	**8**	4	≤0.06
PAM4798	VIM-2	**8**	4	≤0.06	**>64**	>64	0.25	**8**	8	≤0.06
PAM4881	VIM-7	**0.25**	0.125	≤0.06	**4**	2	0.125	**1**	1	≤0.06
PAM4887	IMP-1	**64**	64	1	**>64**	>64	2	**8**	4	0.25
PAM4888	IMP-4	**32**	32	1	**>64**	64	2	**4**	4	0.25
PAM4196	IMP-13	**16**	16	1	**64**	32	2	**1**	1	0.5
PAM4198	IMP-15	**16**	16	0.5	**64**	64	1	**1**	1	0.125
PAM4890	IMP-19	**>64**	32	2	**>64**	>64	4	**1**	1	4
PAM4889	IMP-26	**64**	64	8	**>64**	>64	16	**8**	8	2
PAM4879	CcrA	**16**	16	≤0.06	**4**	4	0.125	**2**	2	≤0.06
PAM4883	GIM-1	**8**	8	0.25	**32**	32	0.5	**32**	16	0.5
PAM4885	SPM-1	**>64**	>64	64	**>64**	>64	>64	**64**	64	32
PAM4880	L1	**4**	2	1	**64**	64	16	**64**	64	32

a 1A-CRB, class A carbapenemase; 2D-CRB, class D carbapenemase; BLIs, beta-lactamase inhibitors; AVI, avibactam; ND, not determined. All BLIs were tested at a fixed concentration of 4 μg/ml. Boldface indicates MIC to antibiotic alone.

**TABLE 3:** MICs of oral antibiotics, ceftibuten, cefpodoxime, and tebipenem in combination with avibactam or QPX7728 against the panel of engineered K. pneumoniae strains producing various cloned beta-lactamases^*a*^

Strain	Beta-lactamase	Class	MIC (μg/ml)								
			Ceftibuten			Cefpodoxime			Tebipenem		
			Alone	With BLIs		Alone	With BLIs		Alone	With BLIs	
				AVI	QPX7728		AVI	QPX7728		AVI	QPX7728
KPM1116	None	None	**≤0.06**	≤0.06	≤0.06	**≤0.06**	≤0.06	≤0.06	**≤0.06**	≤0.06	≤0.06
KPM1033	CTX-M-3	A-ESBL	**0.25**	≤0.06	≤0.06	**32**	≤0.06	≤0.06	**≤0.06**	≤0.06	≤0.06
KPM1031	CTX-M-14	A-ESBL	**2**	≤0.06	≤0.06	**>64**	≤0.06	≤0.06	**≤0.06**	≤0.06	≤0.06
KPM1114	CTX-M-15	A-ESBL	**0.5**	≤0.06	≤0.06	**16**	≤0.06	≤0.06	**≤0.06**	≤0.06	≤0.06
KPM3349	CTX-M-27	A-ESBL	**≤0.06**	≤0.06	≤0.06	**>64**	4	1	**≤0.06**	≤0.06	≤0.06
KPM1924	GES-1	A-ESBL	**0.125**	≤0.06	≤0.06	**4**	≤0.06	≤0.06	**≤0.06**	≤0.06	≤0.06
KPM3735	OXY-6-2	A-ESBL	**≤0.06**	≤0.06	≤0.06	**16**	1	0.5	**≤0.06**	≤0.06	≤0.06
KPM3736	PER-2	A-ESBL	**2**	0.125	≤0.06	**64**	4	0.5	**≤0.06**	≤0.06	≤0.06
KPM3809	PER-4	A-ESBL	**64**	8	≤0.06	**64**	16	≤0.06	**≤0.06**	≤0.06	≤0.06
KPM3258	SHV-5	A-ESBL	**2**	≤0.06	≤0.06	**16**	≤0.06	≤0.06	**≤0.06**	≤0.06	≤0.06
KPM1115	SHV-12	A-ESBL	**1**	≤0.06	≤0.06	**16**	0.125	≤0.06	**≤0.06**	≤0.06	≤0.06
KPM1040	SHV-18	A-ESBL	**0.5**	≤0.06	≤0.06	**16**	≤0.06	≤0.06	**≤0.06**	≤0.06	≤0.06
KPM1112	TEM-10	A-ESBL	**0.25**	≤0.06	≤0.06	**8**	≤0.06	≤0.06	**≤0.06**	≤0.06	≤0.06
KPM1066	TEM-26	A-ESBL	**≤0.06**	≤0.06	≤0.06	**4**	≤0.06	≤0.06	**≤0.06**	≤0.06	≤0.06
KPM3810	VEB-1	A-ESBL	**8**	≤0.06	≤0.06	**8**	0.125	≤0.06	**≤0.06**	≤0.06	≤0.06
KPM3812	VEB-2	A-ESBL	**32**	0.125	≤0.06	**16**	0.25	≤0.06	**≤0.06**	≤0.06	≤0.06
KPM3264	GES-19	A-ESBL	**0.5**	≤0.06	≤0.06	**>64**	1	0.25	**0.125**	≤0.06	≤0.06
KPM1113	KPC-2	A-CRB	**2**	≤0.06	≤0.06	**16**	≤0.06	≤0.06	**0.5**	≤0.06	≤0.06
KPM1049	KPC-3	A-CRB	**0.25**	≤0.06	≤0.06	**2**	≤0.06	≤0.06	**0.25**	≤0.06	≤0.06
KPM2646	BKC-1	A-CRB	**0.25**	≤0.06	≤0.06	**16**	≤0.06	0.125	**0.125**	≤0.06	≤0.06
KPM2738	FRI-1	A-CRB	**1**	0.125	0.125	**4**	≤0.06	≤0.06	**0.5**	≤0.06	≤0.06
KPM3266	GES-20	A-CRB	**0.125**	≤0.06	≤0.06	**8**	2	0.5	**0.5**	≤0.06	≤0.06
KPM1030	DHA-1	C	**32**	≤0.06	≤0.06	**16**	≤0.06	≤0.06	**≤0.06**	≤0.06	≤0.06
KPM1045	CMY-2	C	**64**	0.125	≤0.06	**64**	0.125	≤0.06	**≤0.06**	≤0.06	≤0.06
KPM1054	FOX-5	C	**16**	0.125	≤0.06	**>64**	2	≤0.06	**≤0.06**	≤0.06	≤0.06
KPM1956	P99	C	**64**	0.25	0.125	**>64**	0.25	≤0.06	**≤0.06**	≤0.06	≤0.06
KPM3352	MIR-1	C	**64**	0.5	≤0.06	**>64**	8	2	**≤0.06**	≤0.06	≤0.06
KPM1932	NDM-1	B	**>64**	>64	0.5	**>64**	64	2	**32**	32	≤0.06
KPM1935	VIM-1	B	**16**	32	≤0.06	**>64**	>64	0.125	**2**	2	≤0.06
KPM1906	VIM-2	B	**16**	16	≤0.06	**32**	32	≤0.06	**2**	2	≤0.06
KPM1902	VIM-7	B	**8**	8	≤0.06	**16**	8	≤0.06	**0.125**	≤0.06	≤0.06
KPM1996	IMP-1	B	**64**	64	0.25	**64**	64	0.25	**0.5**	0.25	≤0.06
KPM1997	IMP-4	B	**64**	64	0.5	**64**	>64	0.5	**1**	0.5	≤0.06
KPM3256	IMP-15	B	**32**	32	0.25	**32**	32	0.5	**0.25**	0.125	≤0.06
KPM1910	GIM-1	B	**64**	64	32	**>64**	64	64	**4**	4	1
KPM3260	CcrA	B	**64**	64	0.5	**64**	64	1	**1**	0.5	≤0.06

a 1A-CRB, class A carbapenemase; 2D-CRB, class D carbapenemase; BLIs, beta-lactamase inhibitors; AVI, avibactam. All BLIs were tested at a fixed concentration of 4 μg/ml. Boldface indicates MIC to antibiotic alone.

QPX7728 enhanced the potency of multiple beta-lactams against all strains producing class A extended-spectrum beta-lactamases (ESBLs; CTX-M, SHV, TEM, GES, VEB, PER, OXY) and carbapenemases (KPC-2, KPC-3, GES-20, NMC-A, SME-2, VCC-1, SFC-1, FRI-1). In the absence of QPX7728, the antibiotic MIC values ranged widely, depending on the beta-lactamase, and often were >64 μg/ml. When MICs were determined in combination with QPX7728 at 4 μg/ml, the MIC values against the majority of strains were generally the same as or less than those seen for the control strains containing the vector alone (≤0.06 to 0.25 μg/ml), consistent with the complete inhibition of beta-lactamase activity by QPX7728. In the case in which QPX7728 was combined with cefpodoxime, there were several strains with class A enzymes (CTX-M-27, OXA-6, PER-4, GES-19, GES-20) that showed a significant (32- to >256-fold effect) but incomplete reversion of resistance (resulting MICs, 0.25 to 1 μg/ml versus ≤0.06 μg/ml for the vector-only control). Future studies with purified enzymes will assess whether this can be explained by the decreased activity of QPX7728 against these beta-lactamases when it is tested with the labile agent cefpodoxime (Tables 1 to 3).

QPX7728 also increased the potency of antibiotics against strains that produced class C beta-lactamases both harbored on plasmids (CMY-2, MIR-1, FOX-5, DHA-1) and chromosomally encoded (AmpC from P. aeruginosa, A. baumannii, and Enterobacter cloacae) and that had a wide range of MIC values for the antibiotics alone (often >64 μg/ml); inhibition often reduced the MICs to the level observed with the vector-only control strain, indicating the complete inhibition of various class C enzymes (Tables 1 to 3). The only exception was a somewhat decreased activity of QPX7728 in combination with cefpodoxime for the strain with MIR-1, where QPX7728 lowered the MIC only to 2 μg/ml and not to the value of ≤0.06 μg/ml observed for the vector-only control strain.

QPX7728 reduced the MIC values of several substrate antibiotics against all strains producing class D beta-lactamases, including the carbapenemases OXA-48 and OXA-23/40/58 from *Enterobacteriaceae* and A. baumannii, respectively. QPX7728 potentiation of the activity of multiple antibiotics against OXA carbapenemases from A. baumannii is of particular significance, as the comparator BLIs had poor or no inhibitory activity against OXA enzymes from A. baumannii. Only avibactam significantly restored the potency of piperacillin or meropenem in the OXA-48-producing strain (Tables 1 to 3).

In the assessment of inhibitory activity against MBL-producing strains, all of the enzymes represented in the strains in these panels belonged to subclass B1 (with the exception of L1, the chromosomal beta-lactamase from Stenotrophomonas maltophilia from subclass B3) (20). As expected, none of the comparator BLIs had inhibitory activity against strains expressing the various MBLs. QPX7728 was highly active against strains producing the NDM and VIM MBLs (Tables 1 to 3), in good agreement with the findings of previous biochemical experiments (*K_i_* range, 14 to 55 nM) (11). QPX7728 (4 μg/ml) increased the potency of all tested antibiotics against VIM-producing clones to the level observed for the vector-only control strain. The MICs of the tested antibiotics for the NDM-producing clones in the presence of QPX7728 (4 μg/ml) tended to be somewhat higher than those for the VIM-producing clones; this was particularly evident for ceftazidime and ceftolozane (MICs of the QPX7728-ceftazidime and QPX7728-ceftolozane combinations, 0.12 to 1 μg/ml for the VIM clones and 32 μg/ml for the NDM clones). The ceftazidime and ceftolozane MICs for the VIM-producing strains of P. aeruginosa were in the 64- to 128-μg/ml range, whereas they were in the 1,024- to 2,048-μg/ml range for the NDM-producing clones (data not shown). The higher MICs of ceftazidime and ceftolozane with QPX7728 (4 μg/ml) for the NDM-producing clones (32 μg/ml) suggest that NDM has very potent activity in the hydrolysis of ceftazidime and ceftolozane, with consequent high MIC values and a ca. 4-fold lower inhibitory potency of QPX7728 against NDM than against VIM (IC_50_s, 55 nM versus 14 nM for NDM-1 and VIM-1, respectively) (11). When tested at higher concentrations of QPX7728 (8 μg/ml), the ceftazidime and ceftolozane MIC values were decreased to 4 to 8 μg/ml (data not shown).

Based on the results of the potentiation experiments, QPX7728 was a potent inhibitor of GIM-1 and of CcrA, the MBL from Bacteroides fragilis. QPX7728 also demonstrated inhibitory activity against some but not all MBLs from the IMP subclass; it was more active in antibiotic potentiation activity against the strains producing IMP-1/4/13/15/19 than against the strain producing IMP-26, in good agreement with the findings of previous biochemical experiments (IC_50_s, 0.61 μM versus 4 μM for IMP-1 and IMP-26, respectively) (11). No activity against SPM-1 (which shares 35.5% identity with IMP-1) was detected based on microbiological experiments (no direct biochemical studies have yet been performed). Very weak potentiation activity (not more than 4-fold) and somewhat antibiotic-specific potentiation activity was observed for cefepime, ceftolozane, and piperacillin but not ceftazidime or meropenem against the strain producing L1, the MBL from the B3 subclass (Table 1). In the absence of biochemical data on L1 inhibition by QPX7728, it is not clear whether or not the observed weak potentiation against this strain was due to the direct inhibition of L1 (with a low potency).

**FIG 2:**
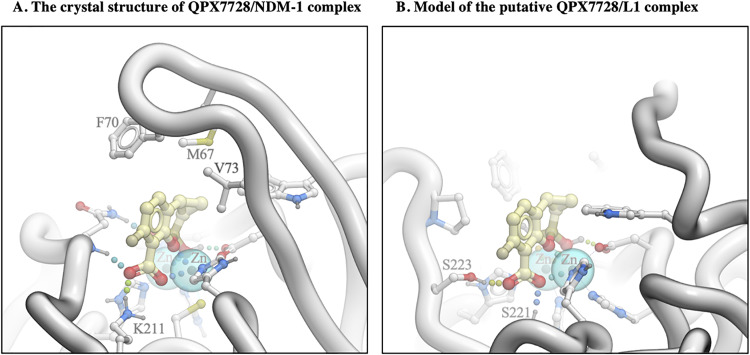
Analysis of interactions of QPX7728 with NDM-1 (A) and L1 (B) beta-lactamases.

Analysis of the crystal structure of the QPX7728/NDM-1 complex (10) suggests that three distinct interactions contribute to the high-affinity binding of the inhibitor: (i) coordination of both zinc ions at the core of the active site by the inhibitor’s boronate hydroxyl and boronate ester oxygen and one of its carboxylic acid’s oxygen atoms, (ii) salt bridge/charge-assisted hydrogen bonds of the QPX7728 carboxylate, and (iii) extensive lipophilic contacts of the QPX7728 phenyl and cyclopropyl rings, largely with the side chains in the L65-V73 loop that caps the active site (Fig. 2A).

In order to elucidate the structural basis for the lack of QPX7728 activity against beta-lactamase L1, we modeled the putative QPX7728/L1 complex by docking. While we found a bound ligand position consistent with coordination of the two metal ions in a manner closely resembling that seen in the available X-ray structures for other MBLs (Fig. 2B), QPX7728 putative binding to L1 did not form a salt bridge due to the lack of a positively charged side chain analogous to K211 in NDM-1). Thus, we hypothesize that QPX7728 would not form comparably extensive hydrophobic interactions due to the lack of the capping loop in L1, where it is truncated to a short hairpin. We reviewed publicly available L1 structures in complex with various ligands to search for potential mobile structural elements in L1 that could serve to cap the active site; none of them exhibited any such structure. Instead, most ligands would form additional hydrophobic interactions further on the periphery of the binding site and beyond the putative QPX7728 binding interface. We therefore conclude that the affinity of QPX7728 to L1 is likely reduced drastically in comparison to its affinity to most other MBLs by the loss of lipophilic interactions with the capping loop and the absence of a salt bridge to K211 or a similar residue. Studies are under way to test this hypothesis and to better understand the specificity of QPX7728 toward particular MBLs.

### Summary.

QPX7728 is a new boronate BLI with an unprecedented spectrum of inhibition of beta-lactamases, including the major representatives of serine and metallo-beta-lactamases from all molecular classes found in infections defined to be urgent and serious threats to public health. Microbiological experiments with an extensive collection of engineered strains with beta-lactamases significantly expanded the information available on the beta-lactamase inhibition profile of QPX7728. They also demonstrated that the broad-spectrum inhibitory activity of QPX7728 observed in cell-free biochemical experiments using purified enzymes translates into enhancement of the potency of multiple i.v. administered (ceftazidime, piperacillin, cefepime, ceftolozane, meropenem) and orally bioavailable (ceftibuten, cefpodoxime, tebipenem) beta-lactams against engineered strains of P. aeruginosa and K. pneumoniae producing various beta-lactamases. QPX7728 inhibits serine class A ESBLs (CTX-M, SHV, TEM, GES, VEB, PER, OXY), carbapenemases (KPC-2/3, GES-20, NMC-A, SME-2, VCC-1, SFC-1, FRI-1), and class C beta-lactamases, both plasmidic (CMY-2, MIR-1, FOX-5, DHA-1) and chromosomally encoded (AmpC from P. aeruginosa, A. baumannii, and E. cloacae). QPX7728 also inhibits class D beta-lactamases, such as oxacillinase OXA-10 and carbapenemases (OXA-48 and OXA-23/40/72/58) from *Enterobacteriaceae* and A. baumannii, respectively. Of note, metallo-beta-lactamase class B, subclass B1, enzymes (NDM, VIM, some IMPs, GIM-1, and CcrA) are inhibited by QPX7728.

QPX7728 has major improvements in its beta-lactamase inhibitory spectrum compared to that of the recently approved agents avibactam (5), vaborbactam (7), and relebactam (21). While QPX7728 and these agents are all potent inhibitors of class A carbapenemases, such as KPC, as well as class C beta-lactamases and, in the case of avibactam, some class D enzymes from *Enterobacteriaceae* (16), none of the existing agents inhibit class D carbapenemases from A. baumannii, such as OXA-23, OXA-24/40, and OXA-58. The existing agents also lack inhibitory activity against various class B metallo-beta-lactamases from the B1 subclass (20), such as NDM, VIM, and IMP. QPX7728 also differs from other investigational beta-lactamase inhibitors in clinical development, such as the avibactam analog durlobactam and the bicyclic boronate taniborbactam (16). The durlobactam spectrum includes OXA enzymes from Acinetobacter, but it does not inhibit class B metallo-beta-lactamases (22, 23). Taniborbactam inhibits some metallo-beta-lactamases, but it lacks inhibitory activity against OXA carbapenemases from Acinetobacter (24, 25). The ultrabroad-spectrum beta-lactamase inhibition profile makes QPX7728 a viable candidate for further development.

## MATERIALS AND METHODS

### Panels of engineered bacterial strains containing cloned beta-lactamases and various combinations of porin and efflux mutations.

The panels of engineered isogenic strains of P. aeruginosa and K. pneumoniae producing over 55 individual beta-lactamases were constructed to study the profile of beta-lactamase inhibition by QPX7728 and comparator BLIs. The construction of recombinant beta-lactamase-producing plasmids (based on a vector plasmid, pUCP24, carrying a gentamicin resistance gene) was described previously (18); the complete set of primers used to amplify the various genes is provided in Table S1 in the supplemental material. Recombinant plasmids were introduced into P. aeruginosa PAM1154 and K. pneumoniae KPM1001 (ATCC 43816) by transformation. PAM1154 lacks MexAB-OprM due to disruption of the *oprM* gene. The use of P. aeruginosa as an isogenic background facilitates the detection of beta-lactamase activity (as an MIC increase) of the low-catalytic-efficiency enzymes that rely heavily on the low permeability of the outer membrane. The use of a strain that lacks efflux pumps ensures no interference from efflux in interpreting the results. KPM1001 is a wild-type strain of K. pneumoniae containing functional genes encoding efflux pumps, such as AcrAB-TolC, and major porins OmpK35 and OmpK36.

### Antimicrobial susceptibility testing.

The bacterial isolates were subjected to broth microdilution susceptibility testing, performed according to Clinical and Laboratory Standards Institute (CLSI) methods (26), using panels prepared in-house. Meropenem was purchased from Sandoz; all other antibiotics were from Sigma-Aldrich. QPC7728 and vaborbactam were synthesized at Qpex Biopharma, Inc., San Diego, CA. Avibactam was purchased from eNovation Chemicals LLC, Bridgewater, NJ, USA, and relebactam was synthesized at Acme Bioscience, Palo Alto, CA, USA, or purchased from AChemBlock, Burlingame, CA, USA.

## Supplementary Material

Supplemental file 1
