# Supplementary material for: Spectrum of Beta-Lactamase Inhibition by the Cyclic Boronate QPX7728, an Ultrabroad-Spectrum Beta-Lactamase Inhibitor of Serine and Metallo-Beta-Lactamases: Enhancement of Activity of Multiple Antibiotics against Isogenic Strains Expressing Single Beta-Lactamases
Source: Antimicrob Agents Chemother. 2020 May 21;64(6):e00212-20. doi: 10.1128/AAC.00212-20 (PMC7269471; doi:10.1128/AAC.00212-20)
Supplement: Supplemental file 1 [file AAC.00212-20-s0001.pdf]

Supplementary Table

Table S1. Source strains and primer sequences used in cloning various beta-lactamase genes

Source strain	Cloned beta-lactamase	Primer name	Primer sequence (5' to 3')	Restriction site added
KP1004	KPC-2	KPC-2-con-F	ACACGAATTC AGGAGGTTATCGTTGATGTCACTGTATCGCCGTC	*Eco*RI
		KPC-2-R1	ACACAAGCTT TACTGCCCGTTGACGCCCAA	*Hind*III
EC1007	KPC-3	KPC-3-con-F	ACACGAATTC AGGAGGTTATCGTTGATGTCACTGTATCGCCGTC	*Eco*RI
		KPC-2-R1	ACACAAGCTT TACTGCCCGTTGACGCCCAA	*Hind*III
SM1000	SME-2	SME-2-conF1	ACACGGATCC AGGAGGTTAATTCTGATGTCAAACAAAG	*Bam*HI
		SME-2-R1	ACACAAGCTT TTAATCAATTGCCTGAATTG	*Hind*III
ECL1004	NMC-A	NMC-conF2	ACGCGAATTC AGGAGGTGTAAAACCATGTCACTTAATGTAAAGC	*Eco*RI
		NMC-A-R1	ACACGGATCC TTATTTAAGGTTATCAATTGC	*Bam*HI
Gene synthesis	BKC-1	BKC1-oSD-F	CCATGATTACGAATTACAGGAAAAATACGGATGACG	In-fusion cloning
		BKC1-R	GGCCAGTGCCAAGCTTCAGGCCTCGGCGGCAATG	In-fusion cloning
Gene synthesis	SFC-1	SFC-1-oSD-F	CCATGATTACGAATTGGTCAAAAAATCAACAATCATG	In-fusion cloning
		SFC-1-R	GGCCAGTGCCAAGCTTTAGAAGCCGATAGACTTTCC	In-fusion cloning
Gene synthesis	VCC-1	VCC-1-oSD-F	CCATGATTACGAATTACTTCTTTAGGAGTTATTTATGAAACGTATT GCTATGTATG	In-fusion cloning
		VCC-1-R	GGCCAGTGCCAAGCTTCACTTTACATTTTCTATTGCA	In-fusion cloning
ECM6625	SHV-5	SHV-12-oF2	ACGCGAATTC GGATGTATTGTGGTTATGCGTTATATTCGCCTGTG	*Eco*RI
		SHV-12-R1	ACACAAGCTT TTAGCGTTGCCAGTGCTCGA	*Hind*III
KP1010	SHV-12	SHV-12-conF2	ACGCGAATTC AGGAGGTTTGTGGTTATGCGTTATATTCGCCTGTG	*Eco*RI
		SHV-12-R1	ACACAAGCTT TTAGCGTTGCCAGTGCTCGA	*Hind*III
KP1012	SHV-18	SHV-12-conF2	ACGCGAATTC AGGAGGTTTGTGGTTATGCGTTATATTCGCCTGTG	*Eco*RI
		SHV-12-R1	ACACAAGCTT TTAGCGTTGCCAGTGCTCGA	*Hind*III
ECM6619	TEM-10	TEM-26-conF1	ACGCGAATTC AGGAGGTGAAGAGTATGAGTATTCAACATTTCCG	*Eco*RI
		TEM-26-R1	ACACAAGCTT TACCAATGCTTAATCAGTGAGGC	*Hind*III
ECM6621	TEM-26	TEM-26-conF1	ACGCGAATTC AGGAGGTGAAGAGTATGAGTATTCAACATTTCCG	*Eco*RI
		TEM-26-R1	ACACAAGCTT TACCAATGCTTAATCAGTGAGGC	*Hind*III
KP1156	CTX-M-2	CTX-M-2-own-F	ACGCGAATTC CTAATAGAGGATTTTTAATGATGACTCAGAGC	*EcoR1*
		CTX-M-2-p24-R	ACACAAGCTT ACCTCGCTCCATTTATTGCA	*HindIII*
EC1008	CTX-M-3	CTX-M-3-oF2	ACGCGAATTC AGAATAAGGAATCCCATGGTTAAAAAATCACTGCG	*Eco*RI

		CTX-M-3-R1	ACACAAGCTT TTACAAACCGTCGGTGACG	*Hind*III
KP1005	CTX-M-14	CTX-M-14-conF1	ACGCGAATTC AGGAGGTTAATTCTGATGGTGACAAAGAGAGTGCA	*Eco*RI
		CTX-M-14-R1	ACACAAGCTT TTACAGCCCTTCGGCGATG	*Hind*III
KP1009	CTX-M-15	CTX-M-15-conF1	ACACGAATTC AGGAGGTTATCGTTGATGGTTAAAAAATCACTGCG	*Eco*RI
		CTX-M-15-R1	ACACAAGCTT TTACAAACCGTTGGTGACGA	*Hind*III
ECL1090	CTX-M-25	CTX-M-25-own-F	ACACGAATTC GTTCAGGGGATTAGGATGATGAGAAA	*EcoR1*
		CTX-M-25-p24-R	ACACAAGCTT ATCACTCCACATGGTGAGTA	*HindIII*
EC1178	CTX-M-27	CTX-M-27-own-F	ACACGAATTC GTATTGGGAGTTTGAGATGGTGACA	*EcoR1*
		CTX-M-27-p24-R	ACACAAGCTT AACCAGTTACAGCCCTTCGG	*HindIII*
Pa1063	GES-1	GES-1-own-F1	ACACGAATTC ATCTCAAGGGATCACCATGCG	*EcoR1*
		GES-1-p24-R1	ACACAAGCTT CTATTTGTCCGTGCTCAGGA	*HindIII*
PA5301	GES-19	GES-1-own-F1	ACACGAATTC ATCTCAAGGGATCACCATGCG	*EcoR1*
		GES-1-p24-R1	ACACAAGCTT CTATTTGTCCGTGCTCAGGA	*HindIII*
PA5301	GES-20	GES-1-own-F1	ACACGAATTC ATCTCAAGGGATCACCATGCG	*EcoR1*
		GES-1-p24-R1	ACACAAGCTTC TATTTGTCCGTGCTCAGGA	*HindIII*
KX1015	OXY-6-2	OXY-6-2-oSD-F	CCATGATTACGAATTCTATCAAGGAGTCAGAGATG	In-fusion cloning
		OXY-6-2-R	GGCCAGTGCCAAGCTCTACAGCCCTTCGGTCACGA	In-fusion cloning
KX1015	PER-2	PER-2-oSD-F	CCATGATTACGAATTTAACAAGGACAGTCGTATGA	In-fusion cloning
		PER-2-R	GGCCAGTGCCAAGCTTCAATCCGGACTCACTGCAG	In-fusion cloning
Gene synthesis	PER-4	PER4-oSD-F	CCATGATTACGAATTCAAAAGGACAATCCGATGAATGTC	In-fusion cloning
		PER4-R	GGCCAGTGCCAAGCTTTAATTTGGGCTTAGGGCAG	In-fusion cloning
KP1498	VEB-1	VEB-1-own-F	ACACGGATCC TAGGAGTACAGACATATGAAAATCGTA	*BamH1*
		VEB-1-p24-R	ACACAAGCTT TGAGCCAGTGTTAGCGGTAG	*HindIII*
Site-directed mutagenesis of VEB-1	VEB-2	VEB1-2fwd	ATTTTCAAAGTTAAGTTGTCAGCTTGAGCATTTGAATACACAATT GTAAAAAATAAACTT	In-fusion cloning
		VEB1-2rev	AAGTTTATTTTTTACAATTGTGTATTCAAATGCTCAAGCTGACAAC TTAACTTTGAAAAT	In-fusion cloning
ECL1122	VEB-3	VEB-oSD-F	CCATGATTACGAATTCAGATAGGAGTACAGACATATGA	In-fusion cloning
		VEB-R	GGCCAGTGCCAAGCTTTATTTATTCAAATAGTAATTCCACGT	In-fusion cloning
PA5433	VEB-9	VEB-oSD-F	CCATGATTACGAATTCAGATAGGAGTACAGACATATGA	In-fusion cloning
		VEB-R	GGCCAGTGCCAAGCTTTATTTATTCAAATAGTAATTCCACGT	In-fusion cloning

PAM1032	PDC-1 (Pa chAmpC)	PA-ampC-own-F	ACACGAATTC CTCATGCAGCCAACGACAAA	*Eco*RI
		PA-ampC-P24-R	ACATAAGCTT TCAGCGCTTCAGCGGCACC	*Hind*III
EC1014	DHA-1	DHA-1-conF1	ACGCGAATTC GGAAGGTTAATTCTGATGAAAAAATCGTTATCTGC	*Eco*RI
		DHA-1-R1	ACACAAGCTT TTATTCCAGTGCACTCAAAATAG	*Hind*III
EC1016	FOX-5	FOX-5-oF2	ACGCGAATTC CACGAGAATAGCCATATGCAACAACGGCGTGCG	*Eco*RI
		FOX-5-R1	ACACAAGCTT TCACTCGGCCAACTGACTCA	*Hind*III
ECL1002	P99	P99-0F2	ACGCGAATTC GACTCGCTATTACGGAAGAT	*Eco*RI
		P99-HIS-R2	ACACAAGCTT TTAGTGGTGATGATGGTGATGCTGTAGCGCCTCGAGGA	*Hind*III
KP1013	CMY-2	CMY-2-oF2	ACGCGAATTC TACGGAACTGATTTCATGATGAAAAAATCGTTATGC	*Eco*RI
		CMY-2-R1	ACACAAGCTT TTATTGCAGCTTTTCAAGAATGC	*Hind*III
ECL1113	MIR-1	MIR-1-own-F	ACACGAATTC TACGGAAGATAACCGATGATGACAA	*EcoR1*
		MIR-1-p24-R	ACACAAGCTT ATGTTTTACTGCAGCGCGTC	*HindIII*
ECL1137	OXA-1	OXA-1-own-F	ACGCGAATTC CCAAACCAATACTTATTATGAAAAACAC	*EcoR1*
		OXA-1-p24-R	ACACAAGCTT GTTGGGCGATTTTGCCATTAG	*HindIII*
KX1000	OXA-2	OXA-2-oF2	ACACGGATCC ATTAAGGAAAAGTTAATGGCAATCCGAATCTTCGC	*Bam*HI
		OXA-2-R1	ACACAAGCTT TTATCGCGCAGCGTCCGAGT	*Hind*III
KX1030	OXA-9	Oxa-9-oSD-F	CCATGATTACGAATTGCGCACAGCGGAGCAATGA	In-fusion cloning
		Oxa-9-R	GGCCAGTGCCAAGCTTCATTTGTTACCCATCAACAC	In-fusion cloning
KP1007	OXA-10	OXA-10-oF2	ACACGAATTC CACCAAGAAGGTGCCATGAAAACATTTGCCGCAT	*Eco*RI
		OXA-10-R1	ACACAAGCTT TTAGCCACCAATGATGCCCT	*Hind*III
EC1062	OXA-48	OXA-48-oF2	ACACGAATTC AAGCAAGGGGACGTTATGCGTGTATTAGCC	*Eco*RI
		OXA-HIS-R1	ACACAAGCTT CTAGTGGTGATGATGGTGATGGGGAATAATTTTTTCCTG	*Hind*III
AB1054	OXA-23	Oxa23-oFWD	ATAAGAATTCATCTGGTGTTTAAAATGAA	*Eco*RI
		Oxa23-REV	CTTAGTCGACTTAAATAATATTCAGCTGTTT	*SalI*
AB1053	OXA-72	Oxa72-oFWD	ATAAGAATTCCCTAACATGAATTTGTAATGA	*Eco*RI
		Oxa72-REV	CTTAGTCGACTTAAATGATTCCAAGATTTTC	*SalI*
AB1057	OXA-58	Oxa58-oFWD	ATAAGAATTCAAATTTTAAAGTTGTATATCATG	*Eco*RI
		Oxa58-REV	CTTAGTCGACTTATAAATAATGAAAAACACC	*SalI*
KP1081	NDM-1	NDM1-oF2	ACACGAATTC GCTGAATAAAAGGAAAACTTG	*Eco*RI
		NDM1-HIS-R1	ACACAAGCTT TCAGTGGTGATGATGGTGATGGCGCAGCTTGTCGGCCA	*Hind*III
KP1292	NDM-7	NDM-1-R	GGC CAG TGC CAA GCT TCA GCG CAG CTT GTC GGC CAT	In-fusion cloning
		NDM-1-oSD-F	CCA TGA TTA CGA ATT CAA TAA AAG GAA AAC TTG ATG GAA TTG C	In-fusion cloning
KP1014	VIM-1	VIM-1-oF2	ACGCGAATTC CCCTATGGAGTCTTGATGTTAAAAGTTATTAGTAG	*Eco*RI
		VIM-1-R1	ACACAAGCTT CTACTCGGCGACTGAGCGATT	*Hind*III
Pa1066	VIM-2	VIM-2-own-F	ACACGAATTC ACAAAGTTATGCCGCACTC	*Eco*RI

		VIM-2-P24-R	ACATAAGCTT CTACTCAACGACTGAGCGAT	*Hind*III
Pa1070	VIM-7	VIM-7-own-F	ACACGGATCC ACAAAGTTATCGCAGTCGG	*Bam*HI
		VIM-7-P24-R	ACATAAGCTT ACTCGGCCACCGGGCGTACTTT	*Hind*III
Pa1068	SPM-1	SPM-1-own-F1	ACACGAATTC TTATCGGAGATCGGAATGAAC	*Eco*RI
		SPM-1-P24-R1	ACACAAGCTT CTACAGTCTCATTTCGCCAA	*Hind*III
Pa1069	GIM-1	GIM-1-own-F	ACACGGATCC AGTTAGAAGGATGATTTCC	*Bam*HI
		GIM-1-P24-R	ACATCTGCAG TTAATCAGCCGACGCTTCAGC	*Pst*I
KP1097	IMP-1	IMP-oF1	ACACGAATTC GTTAGAAAAGGAAAAGTATGA	*EcoRI*
		IMP-R1	CGACGGATCC TTAGAAATTTAGTTACTTGG	*Bam*HI
SM1002	IMP-4	IMP-oF12	ACACGAATTC CCCTAAAACAAAGTTAGAAAAGG	*EcoRI*
		IMP-4-26-clone-R	ACACGGATCC TTAGTTGCTTAGTTTTGATGG	*Bam*HI
Pa1064	IMP-13	PA-IMP-own-F	ACGCGAATTC TAGAAAAGGDWARGTATGAA	*Eco*RI
		PA-IMP-R	ACGCGGATCC GTTAGAAAWTTAGYTACTTGG	*Bam*HI
Pa1067	IMP-15	PA-IMP-own-F	ACGCGAATTC TAGAAAAGGDWARGTATGAA	*Eco*RI
		PA-IMP-R	ACGCGGATCC GTTAGAAAWTTAGYTACTTGG	*Bam*HI
Pa1065	IMP-18	PA-IMP-own-F	ACGCGAATTC TAGAAAAGGDWARGTATGAA	*Eco*RI
		PA-IMP-R	ACGCGGATCC GTTAGAAAWTTAGYTACTTGG	*Bam*HI
ECM6846	CcrA	BF-ccrA-own-F	ACGCGAATTC ATATAAAAGAATAAAATGAAAACAGTATTTATCC	*Eco*RI
		BF-ccrA-R	ACGCAAGCTT CTATGGCTTTGAAGTGCTTTC	*Hind*III
SMM1020	L1	SM-K279a-L1-own-F	ACACGAATTC AAGCGGACGTGGATCATGCGTTTTACCCTGCTCGC	*Eco*RI
		SM-K279a-L1-R	ACATAAGCTT TCAGCGGGTCCCGGCCGTTT	*Hind*III

All source strains are part of the Qpex strain collection. Abbreviations of species that served as a source of cloned genes: KP, *Klebsiella pneumoniae*; SM, *Serratia marcescens*; ECL, *Enterobacter cloacae*, ECM or EC, *Escherichia coli*; KX, *Klebsiella oxytoca*; Pa, *Pseudomonas aeruginosa*; SMM, *Stenotrophomonas maltophilia*.

Sequences corresponding to restriction sites are underlined.

Primer design and experimental procedures for "In-Fusion" cloning were performed according to manufacturer's instructions supplied with "In-Fusion® HD Cloning Plus" kit (TakaraBio, # 638910).
